# Anesthesia management for pericardiectomy- a case series study

**DOI:** 10.1186/s12871-023-02155-4

**Published:** 2023-06-01

**Authors:** Chunxia Shi, Chao Dong, Lan Yao, Nicole Weiss, Hong Liu

**Affiliations:** 1 Department of Anesthesiology, Peking University International Hospital, Beijing, China; 2 Chinese Academy of Medical Sciences, Fuwai Hospital, Beijing, China; 3 Department of Anesthesiology and Pain Medicine, University of California Davis Health, 4150 V Street, Suite 1200, Sacramento, CA 95817 USA

**Keywords:** Constrictive pericarditis, Pericardiectomy, Cardiac surgery, Transesophageal echocardiography, Outcome

## Abstract

**Background:**

Constrictive pericarditis (CP) is an uncommon disease that limits both cardiac relaxation and contraction. Patients often present with right-sided heart failure as the pericardium thickens and impedes cardiac filling. Pericardiectomy is the treatment of choice for improving hemodynamics in CP patients; however, the procedure carries a high morbidity and mortality, and the anesthetic management can be challenging. Acute heart failure, bleeding and arrhythmias are all concerns postoperatively.

**Methods:**

After IRB approval, we performed the retrospective analysis of 66 consecutive patients with CP who underwent pericardiectomy from July 2018 to May 2022.

**Results:**

Most patients had significant preoperative comorbidities, including congestive hepatopathy (75.76%), New York Heart Association Type III/IV heart failure (59.09%) and atrial fibrillation (51.52%). Despite this, 75.76% of patients were extubated within the first 24 h and all but 2 of the patients survived to discharge (96.97%).

**Conclusions:**

Anesthetic management, including a thorough understanding of the pathophysiology of CP, the use of advanced monitoring and transesophageal echocardiography (TEE) guidance, all played an important role in patient outcomes.

## Introduction

Constrictive pericarditis (CP) is characterized by the loss of pericardial elasticity that occurs as the pericardium thickens and calcifies. Over time patients develop limited myocardial relaxation and contraction, reduced cardiac function, and impaired circulation. There are multiple etiologies of CP: tuberculous, infection, postoperative hemorrhage, rheumatic disease, trauma, tumors, mediastinal radiotherapy and idiopathy [1, 2]. While drug therapy is often used to relieve the symptoms, pericardiectomy is the only way to restore normal cardiac physiology in patients with CP [3, 4]. Early pericardiectomy improves the overall clinical outlook and can prevent the development of cardiac cachexia, severe liver dysfunction and myocardial atrophy. A recent study indicates that pericardiectomy has a high safety margin and favorable clinical outcomes for the treatment of CP [5]. In this case series, we retrospectively studied the perioperative anesthetic management and the surgical outcomes in 66 critically ill patients who underwent pericardiectomy.

## Materials and methods

This is a retrospective single center clinical study at a university hospital. The study was approved by the Ethics Committee of Peking University International Hospital and consent was waived by this committee. From July 2018 to May 2022, a total of 66 consecutive patients (46 men and 20 women) who underwent pericardiectomy for CP were analyzed. All the methods were performed in accordance with the Declaration of Helsinki. After IRB approval, the following parameters were collected from the patients’ medical records: age, New York Heart Association (NYHA) cardiac function classification, hypertension, coronary artery disease, diabetes, previous myocardial infarction, pulmonary arterial hypertension, atrial fibrillation (AF), congestive hepatopathy, heart failure (HF), left ventricular ejection fraction (EF), surgical classification, surgical time, cardiopulmonary bypass (CPB) time, aortic cross clamp time, postoperative mechanical ventilation time, length of intensive care unit stay, length of hospital stay (LOS), redo-open heart surgery, blood loss, transfusion, preoperative and postoperative hemoglobin, postoperative complications and perioperative death.

### Statistical analysis

continuous variables were reported as mean ± standard deviation (SD) and compared with a 2-sample t tests. Categorical variables were reported as number and percentage and compared with χ^2^ test. Data management and statistical analyses were conducted with R software (version 4.0.2). A p value of equal or less than 0.05 was considered statistically significant.

## Results

The mean age of the patients was 48.24 years with an age range from 18 to 73 years. The procedures were all performed by the same surgical team and general anesthesia (GA) was utilized in all cases. Patients were induced with midazolam, etomidate, sufentanil or fentanyl and rocuronium. Maintenance anesthesia included sevoflurane, propofol, dexmedetomidine, cisatracurium and sufentanil or fentanyl. In addition to standard monitoring, invasive arterial blood pressures, central venous pressures (CVP), and transesophageal echocardiography (TEE) were utilized. The processed electroencephalogram (pEEG) monitoring was also used to titrate anesthesia level and prevent intraoperative recall.

Preoperatively, 50 patients (75.76%) had signs of congestive hepatopathy, 39 patients (59.09%) had a history of NYHA class III or IV HF and 51.52% of the patients had a diagnosis of AF. Other co-morbidities included coronary artery disease (7.58%), PHTN (13.64%), diabetes mellitus (12.12%), HTN (9.09%) and 1 patient with a previous myocardial infarction (1.52%). The thickness of pericardium before surgery is 7.42 ± 2.31 mm. The CVP before and after pericardiectomy is 14.82 ± 5.26mmHg and 5.20 ± 2.15mmHg, respectively. All patients showed having left ventricular diastolic dysfunction on preoperative transthoracic echocardiography (TTE). The demographic and clinical characteristics are summarized in Table 1.

**Table 1:** Demographics and clinical characteristics of study cohort

Characteristics	N (%)
Age(yr), Mean (SD)	48.24 (14.17)
Female, N (%)	20 (30.30)
Diabetes, N (%)	8 (12.12)
Hypertension, N (%)	6 (9.09)
Coronary heart disease, N (%)	5 (7.58)
Previous myocardial infarction, N (%)	1 (1.52)
Renal insufficiency, N (%)	1 (1.52)
Atrial fibrillation, N (%)	34 (51.52)
Pulmonary arterial hypertension	9 (13.64)
Liver congestion, N (%)	50 (75.76)
Heart failure, N (%)	39 (59.09)
Redo open heart surgery, N (%)	31 (46.97)
NYHA class II, N (%) III, N (%) IV, N (%)	13 (19.70) 41 (62.12) 12 (18.18)
Left atrial diameter (mm), Mean (SD)	47.40 (11.73)
Left ventricular end diastolic diameter(mm), Mean (SD)	39.98 (5.81)
EF (%), Mean (SD)	62.49 (8.68)
Pericardium thickness (mm), Mean (SD)	7.42(2.31)
Preoperative CVP pressure (mmHg),Mean (SD)	14.82(5.26)
Postoperative CVP pressure (mmHg),Mean (SD)	5.20(2.15)

Note: SD: standard deviation; NYHA: New York Heart Association; EF: ejection fraction

Within the study period, 40 patients received an isolated pericardiectomy (60.60%). The remainder of the patients underwent a pericardiectomy in combination with other cardiac procedures, including, valvular surgery (23 cases, 34.85%), coronary artery bypass grafting (1 case, 1.52%), tumor resection (1 case, 1.52%) and pulmonary artery surgery (1 case, 1.52%). 31 cases (46.97%) underwent re-do surgery (Table 2).

**Table 2:** Types of Surgery

Surgical classification	N (%)
Pericardiectomy alone + Mitral valve surgery +Tricuspid valve surgery +Mitral valve and tricuspid valve surgery +Aortic valve and mitral valve surgery +Aortic valve, Mitral valve and tricuspid valve surgery +Mitral valve and tricuspid valve surgery and congenital heart disease correction +Coronary artery bypass grafting +Tumor resection +Pulmonary artery stenosis lysis	40 (60.60) 1 (1.52) 5 (7.58) 12 (18.18) 1 (1.52) 1 (1.52) 3 (4.55) 1 (1.52) 1 (1.52) 1 (1.52)
Redo open-heart surgery Pericardiectomy alone +Tricuspid valve surgery +Mitral valve and tricuspid valve surgery +Aortic valve and mitral valve surgery +Mitral valve and tricuspid valve surgery and congenital heart disease correction	31 (46.97) 20 (30.30) 2 (3.03) 6 (9.09) 1 (1.52) 2 (3.03)

Except for one case, all 40 isolated pericardiectomies were done without the use of CPB. The one case that utilized CPB was a third time redo pericardiectomy with severe preexisting adhesions. For the patients who received a pericardiectomy together with other cardiac procedures, the pericardiectomy portion of the case was performed first without CPB. After completion of pericardiectomy, the other procedures were done on CPB.

There was no difference in the preoperative hemoglobin concentration between the group of patients who had the procedure done on CPB and the group of patients who had the procedure done off-pump; however, the patients who underwent CPB had a lower hemoglobin concentration at end of surgery, more blood loss and a higher rate of transfusion (P < 0.001) when compared to the off-pump group. Inotropes were used in all patients. A total of 50 patients (75.76%) were extubated within 24 h after operation. Both the ICU stay and the LOS were shorter in the off-pump group when compared to the on-pump group, but there was no significant statistical difference (P = 0.070 and P = 0.074, respectively) (Table 3). The incidence of postoperative complications was 57.58%, including infection, re-exploration, acute kidney injury (AKI, based on the Kidney Disease Improving Global Outcomes (KDIGO) criteria), tracheotomy, and low cardiac output (CO). (Table 4).

**Table 3:** Univariate analysis of Non-CPB group and CPB group

Characteristics	Non-CPB Group	CPB Group	P value
N	39	27	
Intraoperative blood loss, (ml), Mean (SD)	190.51 (129.11)	719.29 (409.18)	< 0.001
Blood transfusion, N (%)	0 (0)	9 (33.33)	< 0.001
Intraoperative fluid (ml), Mean (SD)	493.59 (269.06)	1732.85 (875.92)	< 0.001
Intraoperative urine (ml), Mean (SD)	1456.41 (889.20)	1686.67 (805.82)	0.325
Hemoglobin before surgery (g/dl), Mean (SD)	145.15 (18.20)	141.52 (14.67)	0.392
Hemoglobin after surgery (g/dl), Mean (SD)	152.10 (18.81)	114.56 (14.67)	< 0.001
ICU stay (day), Mean (SD)	3.36 (3.39)	7.23 (12.48)	0.070
Length of postoperative stay (day), Mean (SD)	9.79 (5.76)	14.12 (11.82)	0.055
In-hospital stay (day), Mean (SD)	17.26 (6.63)	21.68 (12.78)	0.074

Note: CPB: cardiopulmonary bypass; SD: standard deviation; ml: milliliter

**Table 4:** postoperative complications

Complications	N (%)
Any complication	38 (57.58)
Infection	10 (15.15)
Re-exploration Bleeding Mitral valve and tricuspid valve repair	7 (10.61) 6 (9.09) 1 (1.52)
AKI	7 (10.61)
Re-intubation	5 (7.58)
tracheotomy	4 (6.06)
Low cardiac output	2 (3.03)
New onset AF	2 (3.03)
Unhealing wound	1 (1.52)
Death	2 (3.03)

Note: AKI: acute kidney injury

Transesophageal echocardiography (TEE) showed that there were 6 patients with more than moderate regurgitation in mitral valve and 9 patients in tricuspid valve before pericardiectomy. However, the number was 16 and 19 respectively after pericardiectomy. Total of 17 patients (25.76%) underwent additional valvular repair.

All but two patients survived to discharge (96.97%). Both patients who died wereredo-surgeries with a history of AF and HF prior to surgery. One of the patients developed ventricular fibrillation post-CPB and required an intra-aortic balloon pump (IABP) placement for the reduced EF. The patient died on postoperative day (POD) 1 from refractory ventricular arrhythmias. The other patient developed acute respiratory failure and required prolonged mechanical ventilation. The patient went into ventricular fibrillation on POD 32 and subsequently died.

## Discussion

**Fig. 1:**
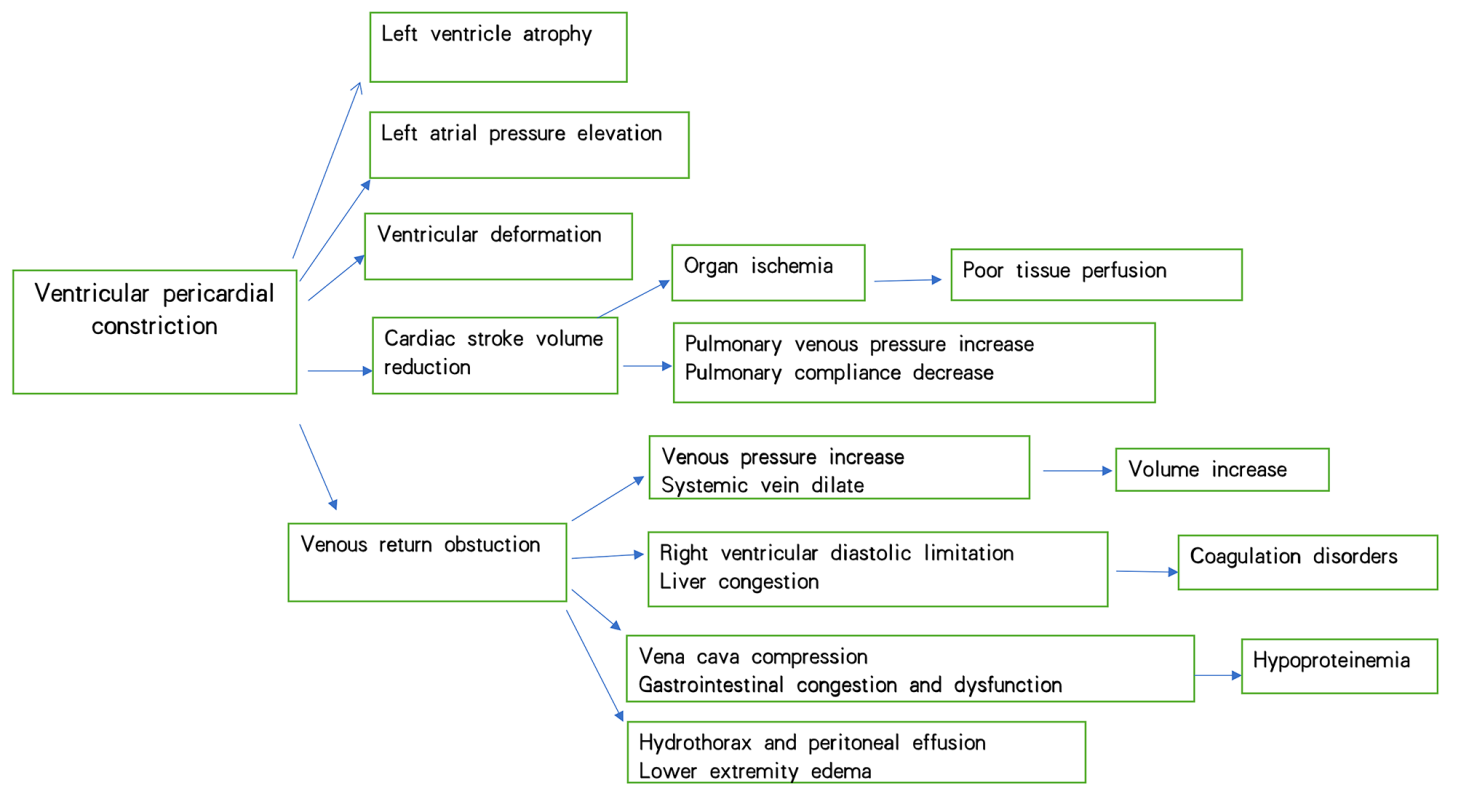
Constrictive pericarditis pathophysiological changes

Constrictive pericarditis is characterized by the pathologic changes of the pericardium that occur over time with chronic inflammation. Pericardial fibrosis, calcification and stiffening leave the heart unable to fully expand and fill, resulting in ventricular constriction. This fundamental pathophysiologic change in diastolic filling leads to right HF and systemic congestion (Fig. 1) [2]. Patients often present with lower extremity edema, poor exercise tolerance and hypoproteinemia. Another important pathologic change of CP is the myocardial atrophy that can occur from myocardial infiltration and fibrosis which not only impairs ventricular diastolic filling, but also affects systolic contraction and further contributes to the development of HF [9].

While pericardiectomy is considered the gold standard for treatment, the physiologic changes associated with CP increase the risk of surgery. Myocardial atrophy and a higher NYHA classification are independent risk factors for a poor prognosis [10]. Hypoproteinemia can affect wound healing and infection rates. Chronic liver stasis can result in hepatic dysfunction and coagulation disorders [6]. When combined with the coagulation dysfunction caused by CPB, the presence of an underlying coagulopathy can significantly increase the surgical risk [7, 8]. A recent meta-analysis with 2,114 patients showed that the operative mortality of CP was 6.9% [11]. Results from other studies also demonstrated that the mortality and morbidity associated with pericardiectomy was significantly higher than that of other cardiac surgeries [12–14]. Despite these statistics, all but two patients in this case series survived to discharge (96.97%) and almost all of these patients had extensive comorbidities (including congestive hepatopathy, HF, and AF) which are known to significantly increase the perioperative mortality [15, 16]. Though many factors can influence patient outcome, anesthetic perioperative management is crucial to achieving good outcomes after pericardiectomy.

### Intraoperative fluid management

Patients undergoing pericardiectomy are at high risk for left HF. When the atrophied left ventricle is freed from the restraint of the scarred pericardium, it can easily be overwhelmed with the sudden increase in volume return from the dilated venous system. Additionally, acute fluid shifts can occur as the venous pressure drops and interstitial fluid is pulled back into the systemic circulation. Meticulous intraoperative fluid management is critical to the overall success of the procedure. A recent study shows that, in patients undergoing pericardiectomy, the total amount of intraoperative fluid significantly correlated with patient outcomes [18]. Minimizing intraoperative fluid infusion and using diuretics from the beginning of surgery were effective in this study. Huang and colleagues indicated that adequate preoperative preparation and a restrictive fluid strategy including the use of diuretics could prevent low CO syndrome and reduce mortality [19].

In this case series, all patients underwent strict preoperative fluid management with a weight loss of 5-10 kg. As most patients were on chronic diuretics, the urine output was meticulously followed during the case. For off-pump pericardiectomies, the target urine output during the procedure was greater than 1000mL. The systolic blood pressure was maintained greater than 90mmHg to ensure adequate renal flow. Low-dose inotropes and, if necessary, administration of vasopressors were used to achieve this goal. Close monitoring of the CVP also helped to guide management decisions. Many patients were noted to have elevated CVPs (15-20mmHg) prior to the surgery. After pericardiectomy, the CVP was kept below 8mmHg. When patients appeared to be at high risk for volume overload, they were placed in a reverse Trendelenberg position. A nitroglycerin infusion was also used to help reduce left ventricular preload if the hemodynamics allowed [17]. Intraoperative TEE monitoring of ventricles can provide a good estimate of preload and guide the fluid management.

### Circulatory support

Postoperatively, it is very important for patients to maintain good myocardial contractility and a fast heart rate [19]. Inotropic agents such as epinephrine or dopamine are the drugs of choice to support cardiac function. All patients were treated with inotropes in this study. Maintaining the heart rate around 100 bpm allows the atrophic heart to recover under an affordable load. While faster heart rates are preferred, severe tachycardia should be avoided and may require treatment with amiodarone when the heart rate exceeds 120 bpm. Although β blockers or non-dihydropyridine calcium channel blockers are often the first-line rate-controlling agents, they need be used judiciously because of their strong negative inotropic effect [20, 21]. When new onset AF affects hemodynamics during pericardiectomy, timely synchronous defibrillation to restore sinus rhythm is vital to maintain circulation stability.

### Perioperative bleeding

Extensive dissection during surgery, preoperative coagulation dysfunction and the use of CPB can all contribute to postoperative bleeding [22]. In our study, 85.71% of patients who required surgical re-exploration had postoperative bleeding. As many CP patients have underlying hepatic dysfunction, Vitamin K should be given prior to the surgery to increase the synthesis of coagulation factors in the liver [23]. The use of an antifibrinolytic, such as tranexamic acid, can also significantly reduce perioperative bleeding and is recommended for the duration of surgery [24–26]. A transfusion algorithm should be used to guide perioperative blood and blood product transfusion.

The utilization of CPB is an independent risk factor for morbidity and mortality after pericardiectomy [15, 16]. It is well known that CPB can lead to an inflammatory response, impaired coagulation function and significant organ dysfunction [27–29]. Although majority of our patients underwent pericardiectomy without CPB, there were still a few patients went on CPB due to combined cardiac surgical procedures instead of the severity of pericarditis. However, the patients who underwent pericardiectomy on-pump lost more blood, required more blood products and had a higher postoperative infection rate when compared to the off-pump group. To minimize the risks of CPB, it is important to complete the pericardiectomy off-pump whenever possible. When CPB is required for concomitant procedures, the pericardiectomy portion of the case should be done prior to institution of CPB.

### Perioperative TEE monitoring

TEE plays an important role during pericardiectomy as it provides for real-time assessment of cardiac structure and function. Perioperative ventricular function and volume status can be assessed and used to guide inotropic support and fluid replacement. Although there may not be significant valvular pathology prior to the surgery, new onset or worsening mitral/tricuspid valve regurgitations are often observed after pericardiectomy which may be caused by increase venous return, ventricular dilation and disfunction and can adversely affect the outcomes if not treated appropriately [30, 31]. Generally, more than moderate mitral and/or tricuspid valvular regurgitations need surgical intervention. In our study, 25.76% of patients underwent valvular repair procedure due to significant worsening of valve regurgitations.

## Conclusions

Constrictive pericarditis is a disease that often leads to severe systemic disease as a result of impaired ventricular relaxation. While pericardiectomy is the treatment of choice, the mortality and morbidity of pericardiectomy is higher than that of other cardiac surgeries. Preoperative comorbidities, including HF and liver disease, are known to affect outcomes and should be taken into account when planning the anesthetic. Anesthesiologists must have a thorough understanding of the physiology of CP to prevent common complications and utilize advanced monitoring, including TEE and CVP, to guide intraoperative management and improve patient outcomes.

## Data Availability

The datasets used and/or analyzed during the current study are available from the corresponding author on reasonable request.

## Abbreviations

AF: atrial fibrillation
AKI: acute kidney injury
CO: cardiac output
CP: constrictive pericarditis
CPB: cardiopulmonary bypass
CVP: central venous pressures
EF: ejection fraction
GA: general anesthesia
HF: heart failure
HTN: hypertension
ICU: intensive care unit
LOS: length of hospital stay
pEEG: processed electroencephalogram
PHTN: pulmonary hypertension
POD: postoperative day
SD: standard deviation
TTE: transthoracic echocardiography
TEE: transesophageal echocardiography
NYHA: New York Heart Association
